# Radioprotective Effects of Gallic Acid in Mice

**DOI:** 10.1155/2013/953079

**Published:** 2013-08-28

**Authors:** Gopakumar Gopinathan Nair, Cherupally Krishnan Krishnan Nair

**Affiliations:** ^1^Department of Radiation Biology, Amala Cancer Research Centre, Thrissur, Kerala 680555, India; ^2^Pushpagiri Institute of Medical Sciences & Research Centre, Thiruvalla, Kerala 689101, India

## Abstract

Radioprotecting ability of the natural polyphenol, gallic acid (3,4,5-trihydroxybenzoic acid, GA), was investigated in Swiss albino mice. Oral administration of GA (100 mg/kg body weight), one hour prior to whole body gamma radiation exposure (2–8 Gy; 6 animals/group), reduced the radiation-induced cellular DNA damage in mouse peripheral blood leukocytes, bone marrow cells, and spleenocytes as revealed by comet assay. The GA administration also prevented the radiation-induced decrease in the levels of the antioxidant enzyme, glutathione peroxidise (GPx), and nonprotein thiol glutathione (GSH) and inhibited the peroxidation of membrane lipids in these animals. Exposure of mice to whole body gamma radiation also caused the formation of micronuclei in blood reticulocytes and chromosomal aberrations in bone marrow cells, and the administration of GA resulted in the inhibition of micronucleus formation and chromosomal aberrations. In irradiated animals, administration of GA elicited an enhancement in the rate of DNA repair process and a significant increase in endogenous spleen colony formation. The administration of GA also prevented the radiation-induced weight loss and mortality in animals (10 animals/group) exposed to lethal dose (10 Gy) of gamma radiation. (For every experiment unirradiated animals without GA administration were taken as normal control; specific dose (Gy) irradiated animals without GA administration serve as radiation control; and unirradiated GA treated animals were taken as drug alone control).

## 1. Introduction

Protection of biological systems from ionizing radiation is of paramount importance in planned as well as unplanned accidental exposures to radiation [1, 2], and development of novel and effective agents to combat radiation damages using nontoxic radioprotectors is of considerable interest in defence, nuclear industry, space travels, and health care, particularly in radiodiagnostics and therapy. Many synthetic as well as natural compounds have been investigated in the recent past for their efficacy to protect the biological systems against the deleterious effects of radiations. They include sulfhydryl compounds, antioxidants, plant extracts, immunomodulators, and other agents [3, 4]. However, the inherent toxicity and side effects of the synthetic agents at the effective radioprotective concentration warranted further search for safer and more effective radioprotectors. Extracts of various plants have been reported to be beneficial for free radical mediated conditions in humans, as they contain compounds having antioxidant activity which can prevent damage induced by reactive oxygen species (ROS) and reactive nitrogen species [5].

In recent years, radioprotecting effects of several natural phytochemicals have been elucidated in short-term *in vitro* tests such as lipid peroxidation, DNA damage, scavenging of free radicals and assays of antioxidant levels, cell survival, and micronuclei induction. However the gold standard for radioprotective activity is the evaluation of 30-days survival in rodents, because the 30-days survival after lethal whole body irradiation clearly indicates the capacity of the agent in test to modulate the recovery and regeneration of the gastrointestinal epithelium and the hematopoietic progenitor cells in the bone marrow, the two most radiosensitive organs that are essential for the sustenance of life [6]. The GI syndrome in mice can be assessed by determining survival up to ten days (measure of GI death) after exposure to comparatively high doses of whole body radiation, whereas hematopoietic syndrome can be assessed by monitoring the survival of irradiated animals up to 30 days after irradiation [7]. Even though there will not be any loss of survival following exposures to low doses of ionizing radiation, alterations do occur in cellular, sub cellular, biochemical and molecular parameters [8]. The deleterious consequences of exposure to low doses of ionizing radiation such as gene mutations and cancer stem from radiation-induced genotoxicity. The ability of a compound to offer protection against genomic insults resulting from the low doses of whole body radiation exposure could be monitored by alkaline comet assay, micronucleus assay, and chromosomal aberration analysis [9].

Naturally occurring polyphenols comprise a wide variety of compounds divided into several classes that occur in fruits and vegetables, wine and tea, and chocolate and other cocoa products [10]. The beneficial effects of phytophenols are mainly attributed to their antioxidant properties, since they can act as chain breakers or radical scavengers depending on their chemical structures [11].

**Scheme 1:**
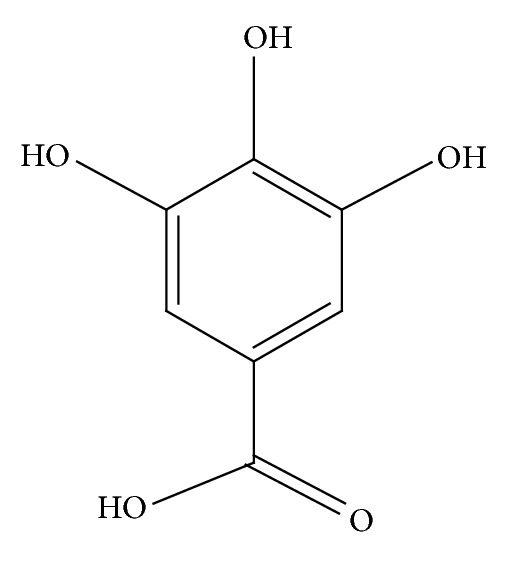
Gallic acid (3,4,5-trihydroxybenzoic acid).

Gallic acid (GA) is a polyhydroxy-phenolic compound (Scheme 1), which can be found in various natural products such as green tea, grapes, strawberries, bananas and many other fruits [12–14]. GA is nontoxic to mammals at pharmacological doses. LD50 dose for GA is 5 g/kg body weight in rats [15]. Gallic acid is known to have anti-inflammatory, antioxidant, free radical scavenging, and radioprotective activity [16–18]. It is employed as an antioxidant in food, in cosmetics, and in the pharmaceutical industry [19, 20]. The present work is focused on the property of GA to protect Swiss albino mice from adverse effects of gamma-radiation such as depletion of tissue antioxidant levels, lipid peroxidation, damages to cellular DNA, and induction of genomic instability in terms of micronuclei and chromosome aberrations in addition to mortality and loss of body weight.

## 2. Materials and Methods

### 2.1. Chemicals

Gallic acid (3,4,5-trihydroxybenzoic acid), thiobarbituric acid (TBA), high melting point agarose and low melting point agarose were procured from Sigma-Aldrich, Inc., St Louis, MO, USA. Na_2_-EDTA was obtained from Sisco Research Laboratories Ltd., Mumbai, India. Silver nitrate, ammonium nitrate, zinc sulphate hepta hydrate, and tungstosilicic acid were from Merck Specialities Pvt. Ltd., Mumbai, India. All other chemicals were of analytical grade procured from reputed Indian manufacturers.

### 2.2. Animals

Male Swiss albino mice weighing 25–28 g were obtained from the Small Animal Breeding Section (SABS), Kerala Agriculture University, Mannuthy, Thrissur, India. They were maintained under standard conditions of temperature and humidity in the Centre's Animal House Facility. The animals were provided with standard mouse chow (Sai Durga Feeds and Foods, Bangalore, India) and water *ad libitum. *All animal experiments were carried out with the prior approval of the Institutional Animal Ethics Committee (IAEC) and were conducted strictly adhering to the guidelines of Committee for the Purpose of Control and Supervision of Experiments on Animals (149/99/CPCSEA) constituted by the Animal Welfare Division of Government of India. 

### 2.3. Exposure to Gamma Radiation

Irradiation was carried out using a ^60^Co-Theratron Phoenix teletherapy unit (Atomic Energy Ltd., Ottawa, Canada) at a dose rate of 1.88 Gy per minute [21].

### 2.4. Effect of Gallic Acid on Whole Body Gamma-Irradiation Induced DNA Strand Breaks in Mouse Peripheral Blood Leukocytes, Bone Marrow Cells, and Spleenocytes

Swiss albino mice were divided into eight groups (*n* = 6 animals/group; total 48 animals), as detailed below:

- 0.2 mL distilled water (oral) + sham irradiation (Not exposed to radiation, normal animals),
- 0.2 mL distilled water (oral) + 2 Gy ^60^Co-*γ*-rays,
- 0.2 mL distilled water (oral) + 4 Gy ^60^Co-*γ*-rays,
- 0.2 mL distilled water (oral) + 8 Gy ^60^Co-*γ*-rays,
- 100 mg/kg body weight GA (oral) + Sham irradiation (Not exposed to radiation, GA Control),
- 100 mg/kg body weight GA (oral) + 2 Gy ^60^Co-*γ*-rays,
- 100 mg/kg body weight GA (oral) + 4 Gy ^60^Co-*γ*-rays,
- 100 mg/kg body weight GA (oral) + 8 Gy ^60^Co-*γ*-rays.

Animals were orally administered with distilled water or with 100 mg/kg body weight GA (dissolved in distilled water, 0.2 mL). One hour after the administration, the animals were exposed to whole body gamma radiation as described above. Immediately after irradiation (within 10 min), the animals were sacrificed, blood and bone marrow were collected, and spleens were excised for alkaline single cell gel electrophoresis or comet assay.

#### 2.4.1. Alkaline Single Cell Gel Electrophoresis

Cellular DNA damage in the mouse tissue samples was measured as single strand breaks using alkaline single cell gel electrophoresis (comet assay) as described earlier [21, 22]. After electrophoresis the slides were dried and subjected to silver staining [23]. The comets were visualised using compound light microscope, images were captured, and a minimum of 50 comets per slide for a group were analysed using the software “CASP” which gives the parameters—% DNA in tail, tail length, tail moment, and Olive tail moment (OTM)—directly [24]. The parameter tail moment (TM) is the product of tail length and the fraction of total DNA in the tail, and OTM is defined as the fraction of tail DNA multiplied by the distance between the profile centres of gravity for DNA in head and tail. OTM incorporates a measure of both the smallest detectable size of migrating DNA (reflected in the comet tail length) and the number of relaxed/broken pieces (represented by the intensity of DNA in the tail) [9]. Results are given as mean ± SD.

### 2.5. Enhancement of Cellular DNA Repair in Mouse Blood Leukocytes by Gallic Acid *In Vivo*

The effect of administration of GA on DNA repair *in vivo* was determined by examining the comet parameters of the blood leukocytes of mice at different post-irradiation intervals. To study the repair of gamma radiation-induced DNA lesions, *in vivo*, blood was collected by tail veining from whole body 4 Gy gamma-irradiated animals at different time intervals. Immediately after tail vein blood collection at 0 time point, the animals were administered with GA or distilled water and blood samples were drawn at 15, 30, 45, 60, and 120 minutes. Alkaline single cell gel electrophoresis or comet assay was performed on these cells.

### 2.6. Effect of Gallic Acid on Gamma-Radiation Induced Micronuclei Formations in Peripheral Blood of Whole Body Irradiated Mice

Animals were administered with GA or distilled water. One hour after administration, the animals were exposed to whole body 2 Gy gamma-radiation. The micronucleus assay with mouse peripheral blood reticulocytes as reported by Hayashi et al., [25], using acridine orange (AO) coated slides, was carried out to evaluate the chromosomal damage. From each of the mice in each treatment group, 5 *μ*L of peripheral blood was collected from the tail without any anticoagulant at 24 and 48 hr of irradiation on to acridine orange coated slides, covered immediately with coverglass, and these slides were allowed to stand for a few hours or overnight in refrigerator to allow cells to settle and to maximize staining. The slides were observed under a blue excitation (488 nm) and a yellow to orange barrier filter (515 nm). The slides were observed for 2000 reticulocytes of peripheral blood (identified by their reticulum structure with red fluorescence) and % of micronucleated (round in shape with a strong yellow-green fluorescence) reticulocytes were scored.

### 2.7. Effect of Gallic Acid on Radiation-Induced Chromosome Aberrations in Whole Body Gamma-Irradiated Mice

Animals were administered with 100 mg/kg bwt of GA or distilled water. One hour after administration, the animals were exposed to whole body 2 Gy gamma-irradiation. At 22 hr after irradiation all the animals were injected i.p. with 2 mg/kg body weight colchicine and sacrificed 2 hr later by cervical dislocation. Both femurs were dissected out and metaphase plates were prepared by air-drying method [26]. Briefly, bone marrow from the femur was aspirated, washed in saline, treated hypotonically (0.565% KCl), fixed in methanol : acetic acid (3 : 1), spread on clean slides, and stained with 4% Giemsa. Chromosomal aberrations were scored under light microscope. A total of 500 metaphases were scored per animal. Different types of aberrations like breaks, rings, and dicentrics as well as cells showing polyploidy, pulverization, and severe damage (SDC, cells with 10 or more aberrations of any type) were scored. Data are presented as mean ± SD.

### 2.8. Effect of Gallic Acid on Endogenous Spleen Colony Formation in Whole Body Gamma-Irradiated Animals

Animals were administered with 100 mg/kg bwt of GA, and 1 hour after drug administration, the animals were exposed to a sublethal dose of 6 Gy whole body gamma-radiation. The administration of GA was continued for 5 days following radiation exposure. The animals were sacrificed on the twelfth day after irradiation by cervical dislocation and the spleen was excised out and fixed in Bouin's solution containing 1.2% saturated picric acid, 30%–40% formalin, and glacial acetic acid in the ratio 15 : 5 : 1, and the spleens were analyzed for colony formations [27]. 

### 2.9. Effect of Gallic Acid on Whole Body Gamma-Irradiation Induced Oxidative Stress and Lipid Peroxidation in Liver, Kidney, and Brain Tissues of Mice

The animals (*n* = 6) administered with 100 mg/kg body weight GA and exposed to various doses (0–8 Gy) of whole body gamma radiation were taken for this study. After 24 hours following irradiation, the animals were sacrificed by cervical dislocation, and liver, kidney, and brain tissues were excised and washed with ice-cold phosphate buffered saline (PBS, 0.2 M, pH 7.4). Tissue homogenates (10%) were prepared in PBS, status of antioxidant enzymes such as GSH (reduced glutathione) and glutathione peroxidase (GPx) was analysed, and radiation-induced damage to membrane was assessed as peroxidation of membrane lipids by analyzing the presence of thiobarbituric acid reacting substance (TBARS) [28]. GSH was expressed as nmoles/mg protein. It may be noted that the concentration of nonprotein thiols in terms of GSH is measured in GSH assay (GSH is taken as standard to quantitate thiols) [29]. In GPx assay, glutathione peroxidise degrades H_2_O_2_ in the presence of glutathione and the remaining GSH is measured, values expressed as U/mg homogenate protein [30]. The lipid peroxidation values are expressed as nanomoles of malondialdehyde (MDA) (using 1,1,3,3-tetraethoxy propane as standard) per mg homogenate protein. Protein was estimated by the method of Lowry et al., [31].

### 2.10. Effect of Administration of Gallic Acid on Whole Body Gamma-Irradiation Induced Lethality and Alterations in Body Weight

Swiss Albino mice were divided into 4 groups, each group comprising 10 animals (total 40 animals), and were treated as detailed below:

- 0.2 mL distilled water (oral) + sham irradiation + 0.2 mL distilled water (oral) continued for next five days (not exposed to radiation, normal (unirradiated) control animals),
- 0.2 mL distilled water (oral) + 10 Gy irradiation + 0.2 mL distilled water (oral) continued for next five days (irradiated control),
- 100 mg/kg body weight GA (oral) + sham irradiation + 100 mg/kg body weight GA (oral), continued for next five days (not exposed to radiation, unirradiated GA control),
- 100 mg/kg body weight GA (oral) + 10 Gy irradiation + 100 mg/kg body weight GA (oral) continued for next five days.

GA was administrated in 0.2 mL distilled water. All the animals were provided with standard diet and water *ad libitum *and were observed on a daily basis to record the mortalities, if any, and the body weights of the survivors.

### 2.11. Statistical Analysis

The results are presented as mean ± SD of the studied groups. Statistical analyses of the results were performed using ANOVA with Tukey-Kramer multiple comparison test. 

## 3. Results

### 3.1. Effect of Gallic Acid on Whole Body Gamma-Irradiation Induced DNA Strand Breaks in Mouse Peripheral Blood Leukocytes, Bone Marrow Cells, and Spleenocytes

**Figure 1:**
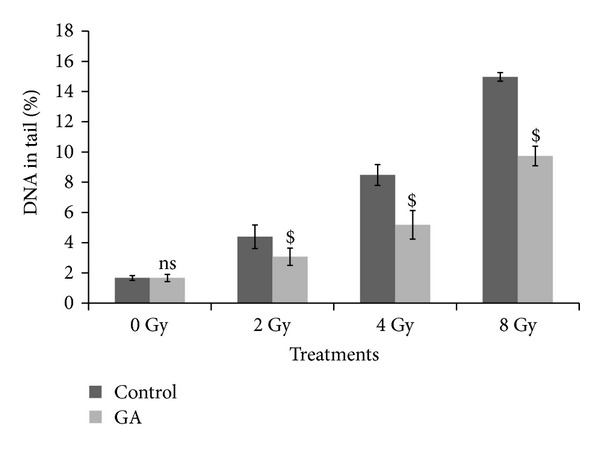
Effect of oral administration of GA on DNA damage in murine blood leukocytes induced by whole body exposure to gamma radiation (0–8 Gy) analyzed by alkaline comet assay. The percentage DNA in tail, presented as mean ± SD (ns indicates not significant and $ indicates *P* < 0.001, when compared with respective controls).

**Figure 2:**
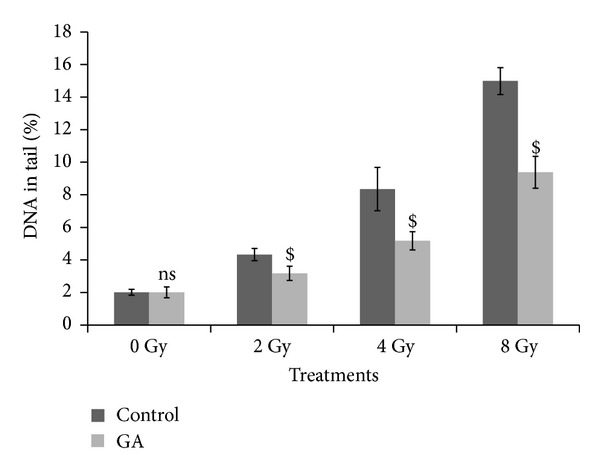
Effect of oral administration of GA on DNA damage in murine bone marrow cells induced by whole body exposure to gamma radiation (0–8 Gy) analyzed by alkaline comet assay. The percentage DNA in tail, presented as mean ± SD (ns indicates not significant and $ indicates *P* < 0.001, when compared with respective controls).

**Figure 3:**
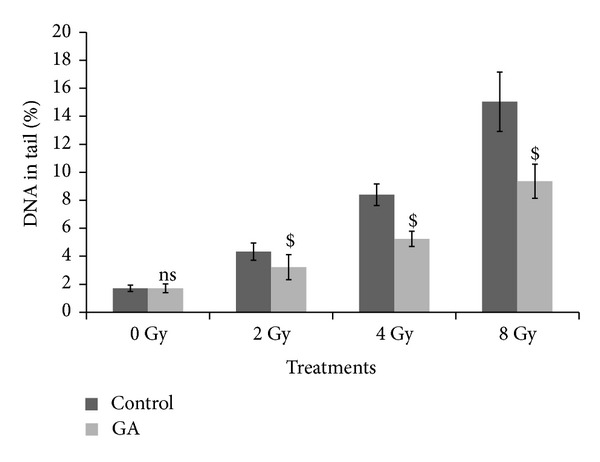
Effect of oral administration of GA on DNA damage in murine spleenocytes induced by whole body exposure to gamma radiation (0–8 Gy) analyzed by alkaline comet assay. The percentage DNA in tail, presented as mean ± SD (ns indicates not significant and $ indicates *P* < 0.001, when compared with respective controls).

Figures 1, 2, and 3 illustrate the results of comet assay in peripheral blood leukocytes, bone marrow cells, and spleenocytes of animals subjected to various treatments, represented by the parameter “% DNA in tail.” The other metrics such as tail length, tail moment, and Olive tail moment also brought essentially the same results. Whole body exposure of animals to gamma radiation (2, 4, or 8 Gy) resulted in cellular DNA damage in various tissues as can be evidenced from the increase in the comet parameters. The parameter % DNA in tail of peripheral blood leukocytes increased from normal control value of 1.67 ± 0.16 to 4.40 ± 0.78, 8.48 ± 0.70, and 14.97 ± 0.28 in 2 Gy, 4 Gy, and 8 Gy irradiated animals, respectively. In bone marrow cells the values increased from 2.01 ± 0.18 to 4.31 ± 0.37, 8.35 ± 1.33, and 14.98 ± 0.83 in 2 Gy, 4 Gy, and 8 Gy irradiated animals, respectively. Similarly in spleenocytes the % DNA in tail increased from 1.71 ± 0.27 to 4.33 ± 0.61, 8.39 ± 0.77, and 15.04 ± 2.12 in 2 Gy, 4 Gy, and 8 Gy irradiated animals respectively. The values of other parameters such as tail length, tail moment, and Olive tail moment also were elevated accordingly. The oral administration of GA to mice one hour prior to whole body irradiation significantly prevented the radiation-induced elevation of the comet aparameters as GA protected the cellular DNA in these tissues. These results thus provide compelling evidence to suggest that GA protected cellular DNA from the radiation-induced damages, under *in vivo* conditions.

### 3.2. Enhancement of Cellular DNA Repair in Mouse Blood Leukocytes by Gallic Acid *In Vivo*

**Figure 4:**
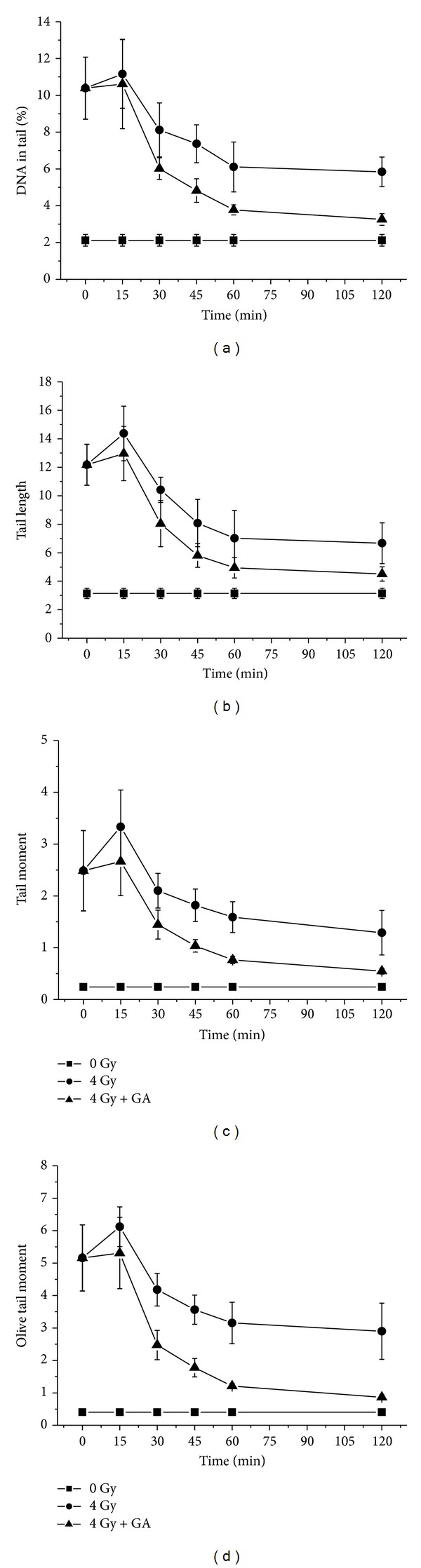
Effect of GA in DNA repair on blood leukocytes of mice exposed to 4 Gy whole body gamma radiation in terms of decrease in comet parameters—% DNA in tail, tail length, tail moment, and Olive tail moment are presented as mean ± SD.

It can be seen from the results presented in Figure 4, that the exposure of animals to 4 Gy gamma-radiation resulted in increase in comet parameters due to radiation-induced cellular DNA damage and the administration of GA to mice, immediately (within 10 min) after whole body radiation exposure, resulted in decrease in these comet parameters at a faster rate compared to the irradiated controls. At 2 h after irradiation in GA administered animals, the various comet parameters such as % DNA in tail, tail length, tail moment, and Olive tail moment were brought down from 10.39 ± 1.69, 12.17 ± 1.44, 2.49 ± 0.77 and 5.16 ± 1.02 to 3.25 ± 0.32, 4.51 ± 0.50, 0.55 ± 0.06, and 0.86 ± 0.07, respectively, while in irradiated controls the respective parameters remained at 5.84 ± 0.8, 6.67 ± 1.43, 1.29 ± 0.43, and 2.9 ± 0.87, suggesting of enhanced repair in GA treated group. It can be surmised from the data presented in the figure that the comet parameters decreased at a faster rate, starting at 15 min after irradiation, in the GA treated animals than in irradiated control animals. Thus the results indicated that post-irradiation administration of GA to mice significantly enhanced the rate of cellular DNA repair process in blood leukocytes under *in vivo* conditions. 

To quantify the efficiency of the cells to repair and rejoin strand breaks in DNA, a relation—cellular DNA repair index (CRI)—was derived based on the comet parameters [21]. CRI for a particular comet parameter is defined as the percentage decrease from the initial value of the parameter due to repair:

(1) $$\begin{array}{c} \text{CRI}=\left[1-\left(\frac{\text{Comet}\;\text{parameter}\;\text{at}\;\text{time}\;t}{\text{Comet}\;\text{parameter}\;\text{at}\;\text{initial}\;\text{time}\;t_{0}}\right)\right]\times 100. \end{array}$$

**Figure 5:**
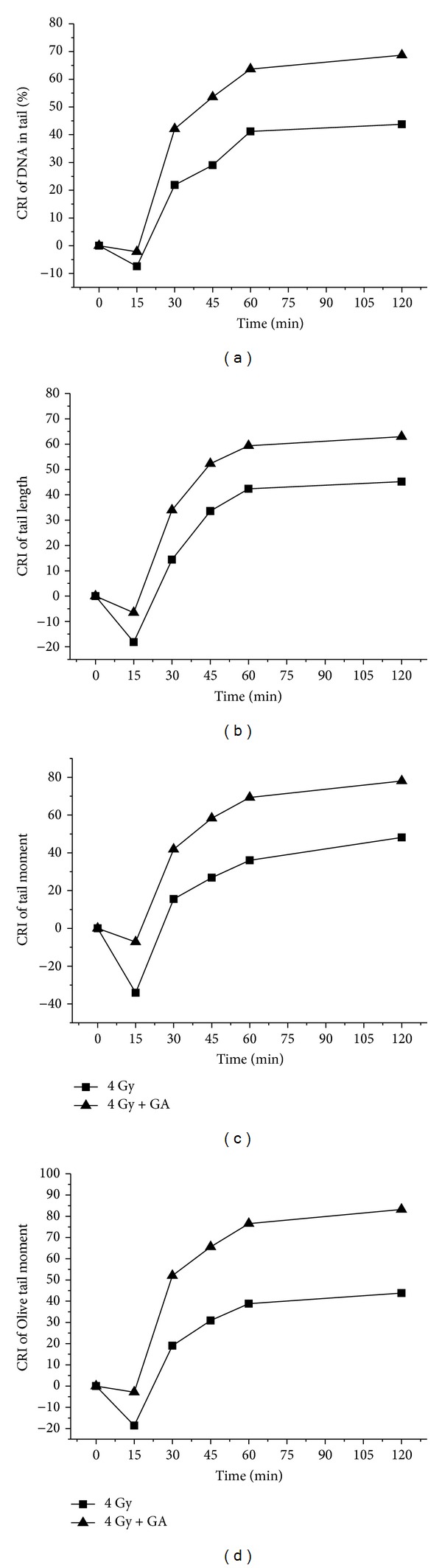
The DNA repair enhancement by GA expressed as cellular DNA repair index (CRI).

As the rate of decrease in the comet parameters is attributed to cellular DNA repair, the efficiency of the cells to repair DNA strand breaks following different treatments can be quantified by determining the CRI. The data on the CRI determined from the comet parameters of mouse peripheral blood leukocytes following 4 Gy whole body gamma-irradiation and postoral administration with 100 mg/kg body weight GA is presented in Figure 5. The CRI increase reflects the disappearance of DNA strand-breaks or DNA repair. In irradiated and GA administered groups, the CRI increased with time at a faster rate than the untreated and irradiated control group. Thus, from the figure it can be observed that GA significantly enhanced cellular DNA repair efficiency.

### 3.3. Effect of Gallic Acid on Gamma-Radiation Induced Micronuclei Formations in Peripheral Blood Reticulocytes of Whole Body Irradiated Mice

**Figure 6:**
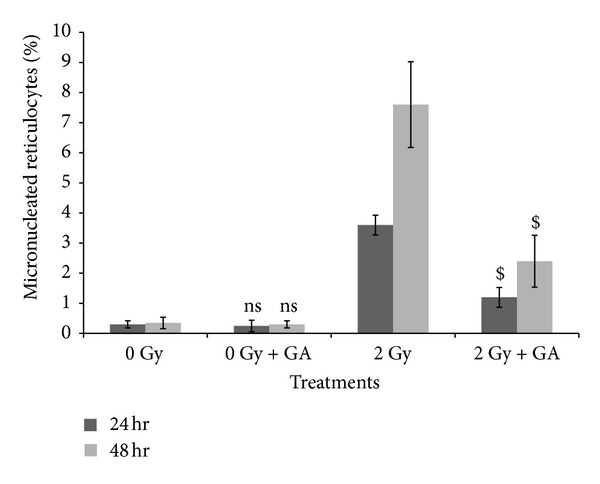
Protection by GA against gamma-radiation (2 Gy) induced micronuclei formation in mice peripheral blood reticulocytes *in vivo*. 2000 reticulocytes of peripheral blood were observed and % of micronucleated reticulocytes was scored. Each point represents the mean ± SD (ns indicates not significant and $ indicates *P* < 0.001, when compared with respective controls).

Whole body exposure of animals to 2 Gy gamma-radiation induces micronucleated reticulocytes in peripheral blood as a result of genomic damage in hematopoietic system. From the data presented in Figure 6 it can be seen that 3.60 ± 0.33 and 7.6 ± 1.42 micronucleated reticulocytes per 100 cells were formed at 24 and 48 hours of post irradiation. The administration GA 100 mg/kg bwt significantly decreased the frequency of micronucleated reticulocytes to 1.2 ± 0.32 and 2.4 ± 0.86 at 24 and 48 hours, respectively. 

### 3.4. Effect of Gallic Acid on Radiation-Induced Chromosome Aberrations in Whole Body Gamma-Irradiated Mice

Sham treated control showed only a few aberrant cells while radiation treatment (whole body 2 Gy radiation) produced a significant increase in the percent aberrant cells. A corresponding increase was found in all the individual aberrations. Treatment of mice with GA at 100 mg/kg bwt resulted in significant decrease in the percent aberrant cells and number of aberrations per cell compared to the control irradiated group. There was a decrease in all types of aberrations, as well as polyploidy and cells with pulverisation (Table 1).

### 3.5. Effect of Gallic Acid on Endogenous Spleen Colony Formation in Whole Body Gamma-Irradiated Animals

**Figure 7:**
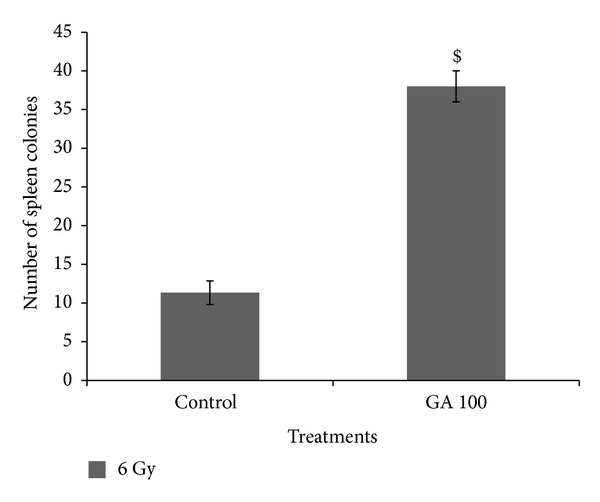
Effect of gallic acid on endogenous spleen colony formation in whole body gamma-irradiated animals (*n* = 4; experiment was repeated twice). ^$^ *P* < 0.001 when compared with the irradiated control.

Formation of endogenous spleen colonies is an index of hematopoietic stem cell proliferation. The administration of 100 mg/kg body weight GA significantly enhanced the spleen colony formation in animals exposed to a sublethal 6 Gy whole body gamma radiation, depicted in Figure 7. The control irradiated animals developed an average of 11.33 ± 1.53 colonies, whereas GA treated group developed 38 ± 2 colonies (*P* < 0.001). 

### 3.6. Effect of Gallic Acid on Whole Body Gamma-Irradiation Induced Oxidative Stress and Lipid Peroxidation in Liver, Kidney, and Brain Tissues of Mice

**Figure 8:**
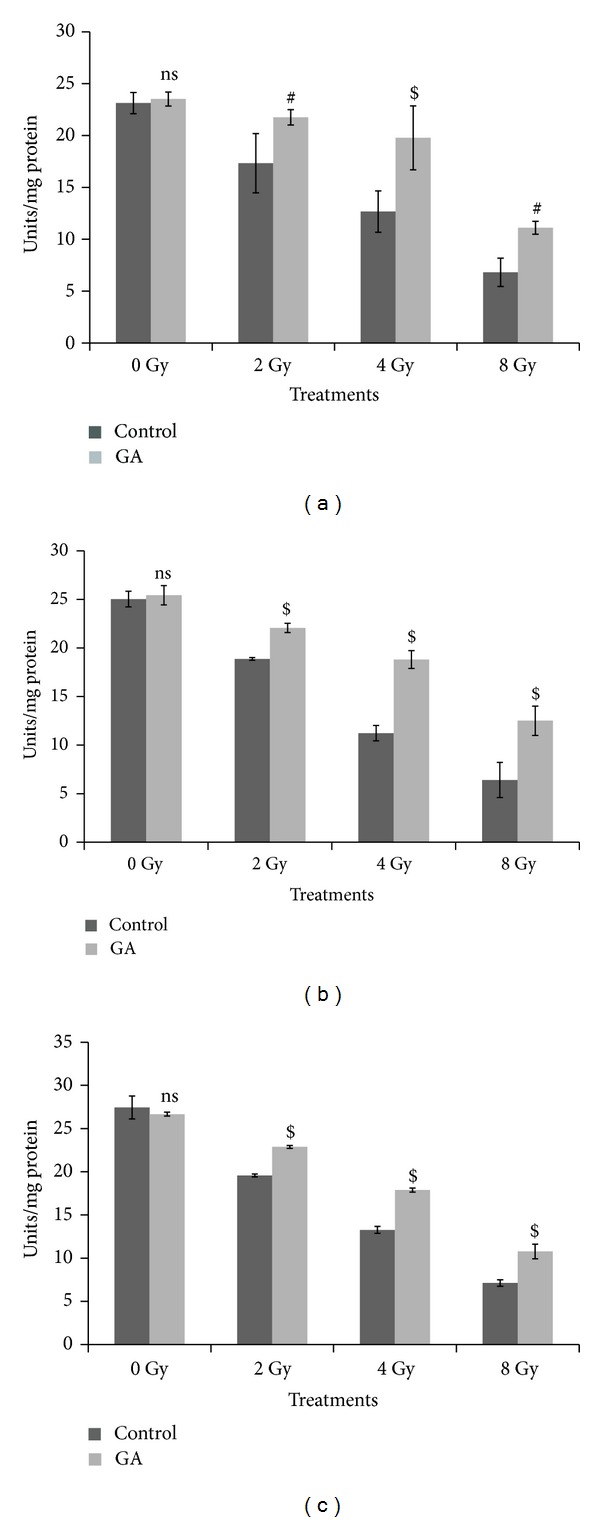
Changes in the GPx levels expressed as units/mg protein in whole body irradiated mice (*n* = 6) (radiation doses 2 Gy, 4 Gy, and 8 Gy) with and without oral administration of GA (100 mg/kg body weight) in liver (a), kidney (b), and brain (c) homogenates (ns indicates not significant, # indicate *P* < 0.01, and $ indicates *P* < 0.001 when compared with respective control).

Activities of the antioxidant defense system such as GPx and levels of GSH were decreased considerably in all tissue types following whole body gamma-irradiation (2–8 Gy).  Figure 8 gives the results on the measurement of GPx levels in the liver, kidney, and brain tissues of whole body gamma irradiated (2, 4, and 8 Gy doses) mice. The GPx levels were decreased from 23.12 ± 1.02 to 17.33 ± 2.86, 12.67 ± 1.99, and 6.82 ± 1.36 in liver tissue; from 25.03 ± 0.80 to 18.87 ± 0.13, 11.23 ± 0.79, and 6.40 ± 1.81 in kidney; and from 27.45 ± 1.32 to 19.58 ± 0.16, 13.28 ± 0.39, and 7.12 ± 0.37 in brain of 2, 4, and 8 Gy irradiated animal groups, respectively. Oral administration of GA (100 mg/kg body weight) one hour before 2, 4, and 8 Gy radiation exposure; restored the GPx levels to 21.75 ± 0.74, 19.78 ± 3.08 and 11.10 ± 0.61 in liver tissue (Figure 8(a)); 22.06 ± 0.48, 18.81 ± 0.92, and 12.50 ± 1.52 in kidney (Figure 8(b)); and 22.88 ± 0.17, 17.89 ± 0.23, and 10.78 ± 0.85 in brain tissue (Figure 8(c)), respectively.

**Figure 9:**
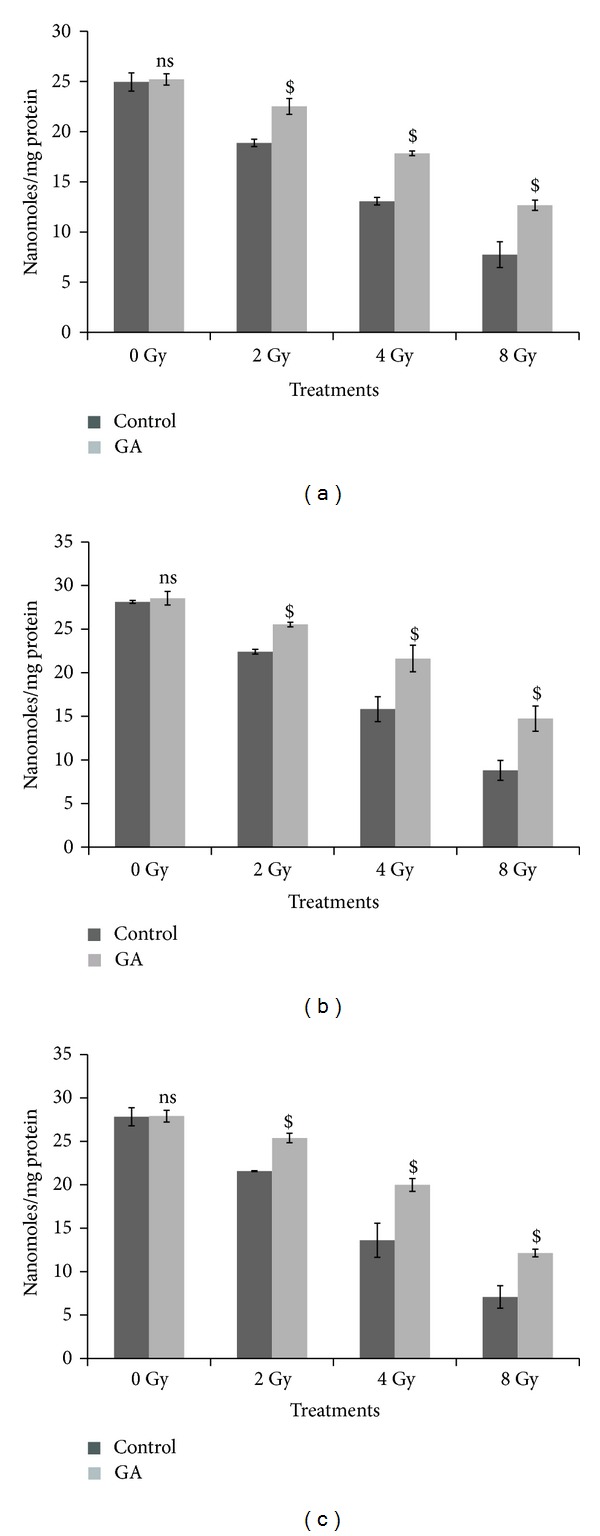
Changes in the GSH levels expressed as nanomoles/mg protein in whole body irradiated mice (*n* = 6) (radiation doses 2 Gy, 4 Gy, and 8 Gy) with and without oral administration of GA (100 mg/kg body weight) in liver (a), kidney (b), and brain (c) homogenates (ns indicates not significant, $ indicates *P* < 0.001 when compared with respective control).

The GSH levels were decreased from 24.96 ± 0.91 to 18.87 ± 0.37, 13.07 ± 0.38, and 7.74 ± 1.29 in the liver tissue of mice exposed to 2, 4, and 8 Gy doses of gamma-radiation, respectively. Administration of 100 mg/kg body weight of GA orally, one hour before 2, 4 and 8 Gy radiation exposure, restored the GSH levels up to 22.52 ± 0.79, 17.84 ± 0.23, and 12.67 ± 0.51, respectively, shown in Figure 9(a).

In the kidney (Figure 9(b)), GSH levels were decreased from 28.12 ± 0.17 to 22.42 ± 0.27, 15.83 ± 1.43, and 8.8 ± 1.13, when the mice were exposed to 2, 4, and 8 Gy doses of gamma radiation respectively and in the case of animals exposed to 2, 4, and 8 Gy doses of radiation, one hour after oral administration of GA (100 mg/kg body weight) the GSH levels were restored to 25.52 ± 0.27, 21.62 ± 1.53, and 14.74 ± 1.44, respectively.

The GSH levels in the brain tissue were decreased from 27.83 ± 1.04 to 21.58 ± 0.06, 13.61 ± 1.96, and 7.08 ± 1.31, when the mice were exposed to 2, 4, and 8 Gy doses of gamma radiation, respectively, and in the case of animals exposed to 2, 4, and 8 Gy doses of radiation, one hour after oral administration of GA (100 mg/kg body weight), the GSH levels were restored to 25.38 ± 0.55, 19.99 ± 0.74, and 12.13 ± 0.44, respectively, depicted in Figure 9(c).

**Figure 10:**
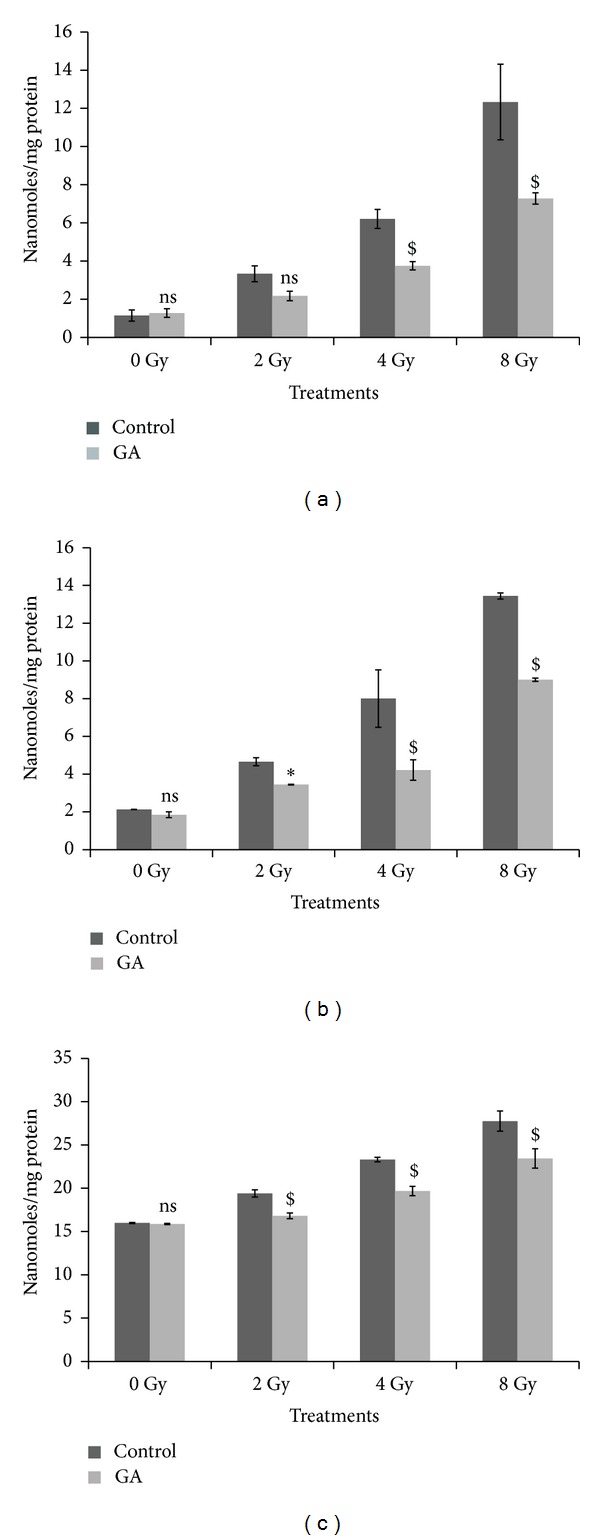
Changes in the lipid peroxidation levels expressed as MDA in nanomoles/mg protein in whole body irradiated mice (*n* = 6) (radiation doses 2 Gy, 4 Gy, and 8 Gy) with and without oral administration of GA (100 mg/kg body weight) in liver (a), kidney (b), and brain (c) homogenates (ns indicates not significant, ∗ indicates *P* < 0.05, and $ indicates *P* < 0.001 when compared with respective control).

The results on the measurement of peroxidation of lipids in terms of MDA or TBARS formed in the liver, kidney and brain tissues of mice exposed to 2, 4, and 8 Gy gamma radiation are depicted in Figure 10. The levels of MDA (nanomoles/mg protein) were increased from 1.15 ± 0.29 to 3.34 ± 0.41, 6.21 ± 0.50, and 12.33 ± 1.98 in liver tissue, from 2.13 ± 0.01 to 4.66 ± 0.21, 8 ± 1.52, and 13.44 ± 0.16 in kidney, and from 15.98 ± 0.06 to 19.40 ± 0.41, 23.31 ± 0.26, and 27.75 ± 1.16 in brain of 2, 4, and 8 Gy irradiated animal groups, respectively. Oral administration of GA (100 mg/kg body weight) one hour before 2, 4, and 8 Gy radiation exposure decreased the MDA levels to 2.18 ± 0.25, 3.75 ± 0.22, and 7.28 ± 0.29 in liver tissue (Figure 10(a)); 3.45 ± 0.02, 4.22 ± 0.54, and 9 ± 0.09 in kidney (Figure 10(b)); and 16.81 ± 0.33, 19.68 ± 0.53, and 23.44 ± 1.12 in brain tissue (Figure 10(c)), respectively.

### 3.7. Effect of Administration of Gallic Acid on Whole Body Gamma-Irradiation Induced Lethality and Alterations in Body Weight

**Figure 11:**
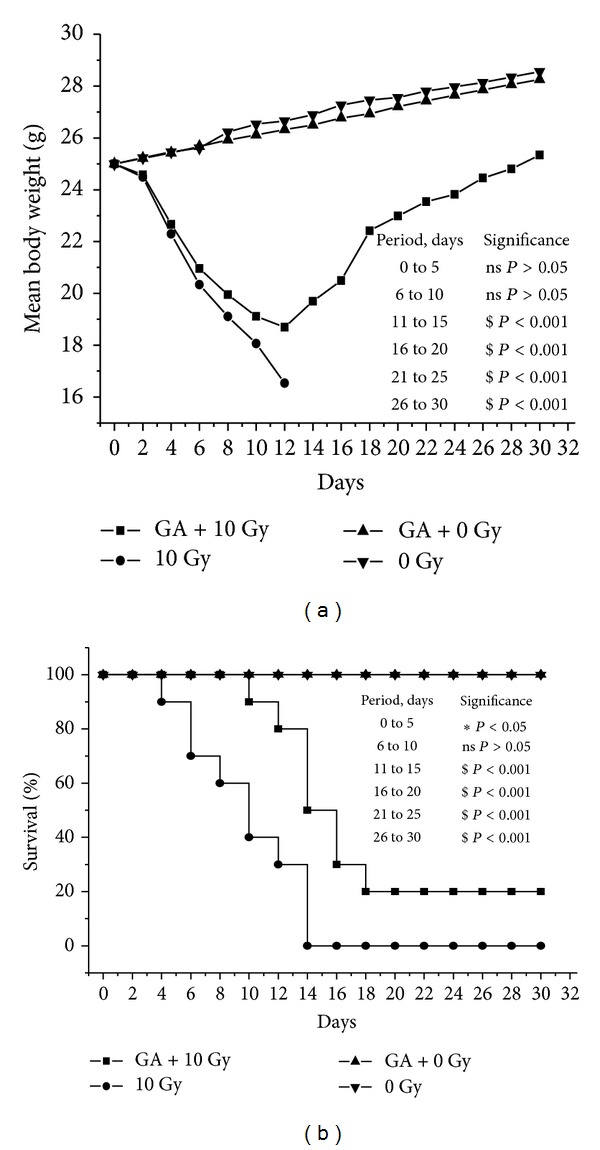
Effect of gallic acid on 10 Gy gamma radiation-induced alterations in body weight (a) and mortality (b). GA (100 mg/kg body weight) was orally administered one hour prior to whole body gamma radiation exposure and continued for next five days (ns indicates not significant, ∗ indicate *P* < 0.05, and $ indicates *P* < 0.001 when GA + 10 Gy group is compared with control irradiated group).

Figures 11(a) and 11(b) represent the changes in body weight and survival of animals following whole body exposure to an acute lethal dose of 10 Gy gamma-radiation. The body weight of the irradiated animals showed a marked decrease following 10 Gy gamma-radiation exposure, while the unirradiated animals showed a gradual increase. The body weight of the animals in the control irradiated group, without GA administration, continued to decrease till all the animals died on the fourteenth day. In the group, where animals exposed to whole body gamma radiation and administered with GA, the body weight of the survivors started recovering after the twelfth day of irradiation.

Animals in the irradiated group started dying from 4th day. On the day 10, the mortality in control irradiated group was 40%, while it was only 10% in the irradiated-GA administered group. On the fourteenth day there was 100% mortality in the irradiated control, while the mortality was only 50% in the irradiated GA administered group till the sixteenth day. On the the eighteenth day, the survival was 20% for this group and there was no further lethality till the thirtieth day.

## 4. Discussion

The toxic effects of ionizing radiation are manifested by direct ionization and through generation of toxic free radicals causing DNA damages such as single strand breaks, double strand breaks, oxidative damage to sugar, and base residues leading to cellular lethality, chromosomal aberration, and mutations [32]. It is well established that ionizing radiation when interacting with living cells, causes a variety of changes depending on the exposed and the absorbed dose, the duration of exposure and the post-irradiation conditions, and the susceptibility of tissues [33]. Efforts to reduce toxicity to normal tissue cells and organs have led to the search for cytoprotective agents. 

Reactive free radicals, such as hydroxyl radicals, hydrogen radicals, and hydrogen peroxide, are the main toxic substances produced by ionizing radiation in living cells. Scavenging and elimination of free radical species constitute the main mechanism of radioprotection by several of these compounds, as they remove and detoxify products of water radiolysis and reactive oxygen species (ROS), before these toxic substances interact with critical macromolecules such as DNA [34]. Unfortunately, most of chemical radioprotectors (AET, WR 2721, and WR 1065) have shown toxic side effects that limit their use in medical practice [35]. Investigations for effective and nontoxic compounds with radioprotecting ability led to increasing interest in naturally occurring antioxidants. The naturally occurring polyphenol, gallic acid, showed significant protection against gamma-radiation induced damages to mammalian system such as genotoxicity, membrane damage, oxidative stress, and radiation-induced weight loss and mortality. 

The cell death caused by radiation is mainly due to deoxyribonucleic acid (DNA) damage. The major DNA damage, due to ionizing radiation are single strand breaks, double strand breaks, DNA-DNA and DNA-protein cross-links, and damages to nucleotide bases. The alkaline comet assay is a powerful and sensitive technique to monitor DNA strand breaks and alkali-labile DNA lesions and is widely used to study genotoxicity, cellular DNA lesions such as single or double strand breaks, apoptosis, and DNA repair [32, 36, 37]. The results of alkaline comet assays performed in tissues, such as peripheral blood leukocytes, bone marrow cells, and spleenocytes of animals exposed to various doses of gamma-radiation, indicated that administration of GA one hour prior to irradiation protected cellular DNA from radiation-induced damages.

The studies on the cellular DNA repair in mice showed that oral administration of GA to mice immediately after 4 Gy whole body gamma-radiation exposure significantly enhanced the rate of cellular DNA repair process. Comet assays performed at various time intervals, to analyze the extent of DNA damage, revealed a faster decrease in the comet parameters in the GA treated animals; the values of CRI were higher in the GA treated group than in the untreated control. The fast decrease in comet parameters and the increased CRI are due to the enhanced rate of repair of DNA strand breaks in the tissues of mice administered with GA after irradiation. Studies have revealed that at physiologically relevant concentrations GA protected DNA and prevented apoptosis and it was postulated that these effects are due to scavenging of radicals and/or induction of DNA repair enzymes [38–40]. In rats the GA has been shown to induce the transcription Nrf2, which controls genes involved in the protection against ROS [41].

The repair system involves removal of DNA lesions such as radiation-induced base modification through a process of incision, excision, replacement, of lesions at the sites of damage and rejoining DNA strands [42]. Misjoined or unrepaired DNA double strand breaks can produce deletions, translocations, and acentric or dicentric chromosomes. Damage to chromosomes is also manifested as breaks and fragments, which appears as micronuclei in the rapidly proliferating cells [43, 44]. Administration of GA prevented the radiation-induced micronuclei formation and chromosome aberrations suggesting protection of radiation-induced DNA damages and efficient DNA repair. The observed increase in endogenous spleen colony would indicate the beneficial effect of GA administration on radioprotection together with support of increased cell proliferation in the hematopoietic tissue.

Gallic acid is known to chelate transition metal ions which are powerful promoters of free radical damage in the human body. In fact, gallic acid was reported to scavenge hydrogen peroxide and inhibit lipid peroxidation. The free gallic acid acts as a potent antioxidant in a living system. The biological activities of gallic acid seem to depend upon its behavior as either an antioxidant or a prooxidant [45–48]. The oral absorption of GA was reported to be fast (*t*_max⁡_: 1.27 ± 0.20 h for acidum gallicum tablets). The sum of GA excreted in urine as unchanged GA and its metabolite, 4OMGA, was 36.4 ± 4.5% for acidum gallicum tablets [15].

Radiation causes decline in antioxidant enzyme levels; administration of GA one hour prior to whole body gamma radiation helped to maintain or restore the antioxidant enzyme GPx in various tissues of Swiss albino mice. GSH or reduced glutathione is an intracellular nonprotein thiol which can directly scavenge free radicals and act as cofactor for enzymes involved in oxidative stress. Exposure to radiation-induced a dose dependant depletion of GSH in various tissues of the animal, and the administration of GA prevented this depletion of GSH. The major damage to membranes by radiation is the oxidation of the membrane lipids by the radiolytic products such as hydroxyl and peroxyl radicals. Lipid peroxidation is a highly destructive process, resulting in the formation of malondialdehyde (MDA), and alters the structure and function of cellular membranes. Administration of GA one hour prior to whole body gamma radiation prevented the radiation-induced increase in MDA levels in the liver, kidney, and brain tissues of the irradiated animals. 

It was also found that the administration of GA could offer protection to mice against lethal dose of whole body gamma-radiation (10 Gy). There was partial recovery of the radiation-induced loss of body weight in survivors. The percent survival was also increased in the GA administered group. On the twelfth day after irradiation there was 80% survival in the GA administered group while it was 30% in the control irradiated group. However, on the fourteenth day there was complete mortality in the control irradiated group, while in the GA administered group 50% survived. In the GA administered group, the percent survival remained at 20% even 18 days after irradiation and there was no further mortality even beyond the thirtieth day.

The present study demonstrated the potential of GA in offering protection against various deleterious effects of ionizing radiations in mammalian system. The increased protecting efficiency of this phytopolyphenol may be due to its free radical scavenging property. Planned human exposures of radiation are warranted in defense, space programmes, nuclear emergencies, and accidents. The present study provides compelling evidence supporting the use of the nontoxic natural polyphenol GA as a radioprotecting drug or food additive under such radiation exposure scenarios.

## Figures and Tables

**Table 1:** Effect of oral administration of gallic acid (GA) on the induction of individual chromosomal aberrations, polyploidy, SDC, pulverization, and number of aberrations per cell in mouse bone marrow cells by whole body gamma irradiation (2 Gy).

Treatments	Breaks (%)	Ring (%)	Dicentric (%)	Aberrations/cell	Pulverisation	Polyploidy	SDC
Normal	1.4 ± 0.89	0 ± 0	1 ± 0.58	0.024 ± 0.015	0 ± 0	0 ± 0	0 ± 0
GA	1.2 ± 1.09^ns^	0 ± 0^ns^	0.8 ± 0.84^ns^	0.02 ± 0.019^ns^	0 ± 0^ns^	0 ± 0^ns^	0 ± 0^ns^
2 Gy	29.8 ± 2.89	8.7 ± 2.59	10.5 ± 0.47	0.49 ± 0.04	2.4 ± 0.81	5.6 ± 0.5	1.6 ± 0.46
GA + 2 Gy	9 ± 0.71^$^	3 ± 0.70^$^	2.5 ± 0.35^$^	0.145 ± 0.02^$^	0 ± 0^$^	1 ± 0^$^	0 ± 0^$^

ns: not significant, $: *P* < 0.001, when compared with respective controls.

## References

[1] Arora R, Gupta D, Chawla R (2005). Radioprotection by plant products: present status and future prospects. *Phytotherapy Research*.

[2] Jagetia GC (2007). Radioprotective potential of plants and herbs against the effects of ionizing radiation. *Journal of Clinical Biochemistry and Nutrition*.

[3] Maisin JR (1998). Bacq and Alexander award lecture chemical radioprotection: past, present and future prospects. *International Journal of Radiation Biology*.

[4] Nair CKK, Parida DK, Nomura T (2001). Radioprotectors in radiotherapy. *Journal of Radiation Research*.

[5] Arora R, Chawla R, Singh S, Govil JN (2006). Bioprospection for radioprotective molecules from indigenous plants. *Recent Progress in Medicinal Plants, Vol 16: Phytomedicine*.

[6] Jagetia GC, Baliga MS (2003). Evaluation of the radioprotective effect of the leaf extract of *Syzygium cumini* (Jamun) in mice exposed to a lethal dose of *γ*-irradiation. *Nahrung—Food*.

[7] Weiss JF, Landauer MR, Gunter-Smith PJ, Hanson WR, Dubois A, King GL, Livengood DR (1995). Effect of radioprotective agents on survival after acute intestinal radiation injury. *Radiation and the Gastrointestinal Tract*.

[8] Sandeep D, Nair CKK (2011). Radioprotection by *α*-asarone: prevention of genotoxicity and hematopoietic injury in mammalian organism. *Mutation Research*.

[9] Ramachandran L, Nair CKK (2011). Protection against genotoxic damages following whole body gamma radiation exposure in mice by lipoic acid. *Mutation Research*.

[10] Manach C, Scalbert A, Morand C, Rémésy C, Jiménez L (2004). Polyphenols: food sources and bioavailability. *American Journal of Clinical Nutrition*.

[11] Rice-Evans C (2001). Flavonoid antioxidants. *Current Medicinal Chemistry*.

[12] Hatano T (1995). Constituents of natural medicines with scavenging effects on active oxygen species—tannins and related polyphenols. *Natural Medicines*.

[13] Okuda T, Yoshida T, Hatano T, Yagi K (1993). Antioxidant phenolics in oriental medicine. *Active Oxygens, Lipid Peroxides, and Antioxidants*.

[14] Tanaka T (1999). Structure, property and function of plant polyphenols. *Foods and Food Ingredients Journal of Japan*.

[15] Shahrzad S, Aoyagi K, Winter A, Koyama A, Bitsch I (2001). Pharmacokinetics of gallic acid and its relative bioavailability from tea in healthy humans. *Journal of Nutrition*.

[16] Kroes BH, van den Berg AJJ, van Ufford HCQ, van Dijk H, Labadie RP (1992). Anti-inflammatory activity of gallic acid. *Planta Medica*.

[17] Gandhi NM, Nair CKK (2005). Protection of DNA and membrane from gamma radiation induced damage by gallic acid. *Molecular and Cellular Biochemistry*.

[18] Lo Scalzo R (2010). Measurement of free radical scavenging activity of gallic acid and unusual antioxidants as sugars and hydroxyacids. *Electronic Journal of Environmental, Agricultural and Food Chemistry*.

[19] Padilla M, Palma M, Barroso CG (2005). Determination of phenolics in cosmetic creams and similar emulsions. *Journal of Chromatography A*.

[20] Ow Y-Y, Stupans I (2003). Gallic acid and gallic acid derivatives: effects on drug metabolizing enzymes. *Current Drug Metabolism*.

[21] Nair GG, Nair CKK (2010). Protection of cellular DNA and membrane from *γ*-radiation-induced damages and enhancement in DNA repair by sesamol. *Cancer Biotherapy and Radiopharmaceuticals*.

[22] Nair GG, Nair CKK (2011). Amelioration of *γ*-radiation induced genomic insult and oxidative stress in whole body irradiated Swiss albino mice by sesamol. *International Journal of Low Radiation*.

[23] Cerda H, Delincee H, Haine H, Rupp H (1997). The DNA ‘comet assay’ as a rapid screening technique to control irradiated food. *Mutation Research/Fundamental and Molecular Mechanisms of Mutagenesis*.

[24] Końca K, Lankoff A, Banasik A (2003). A cross-platform public domain PC image-analysis program for the comet assay. *Mutation Research*.

[25] Hayashi M, Morita T, Kodama Y, Sofuni T, Ishidate M (1990). The micronucleus assay with mouse peripheral blood reticulocytes using acridine orange-coated slides. *Mutation Research*.

[26] Devi PU, Bisht KS, Vinitha M (1998). A comparative study of radioprotection by *Ocimum* favonoids and synthetic aminothiol protectors in the mouse. *British Journal of Radiology*.

[27] Till JE, McCulloch EA (1961). A direct measurement of the radiation sensitivity of normal mouse bone marrow cells. *Radiation Research*.

[28] Buege JA, Aust SD (1978). Microsomal lipid peroxidation. *Methods in Enzymology*.

[29] Moron MS, Depierre JW, Mannervik B (1979). Levels of glutathione, glutathione reductase and glutathione S-transferase activities in rat lung and liver. *Biochimica et Biophysica Acta*.

[30] Hafeman DG, Sunde RA, Hoekstra WG (1974). Effect of dietary selenium on erythrocyte and liver glutathione peroxidase in the rat. *Journal of Nutrition*.

[31] Lowry OH, Rosebrough NJ, Farr AL, Randall RJ (1951). Protein measurement with the Folin phenol reagent. *The Journal of Biological Chemistry*.

[32] Olive PL (1999). DNA damage and repair in individual cells: applications of the comet assay in radiobiology. *International Journal of Radiation Biology*.

[33] Karbownik M, Reiter RJ (2000). Antioxidative effects of melatonin in protection against cellular damage caused by ionizing radiation. *Experimental Biology and Medicine*.

[34] Hosseinimehr SJ (2009). Potential utility of radioprotective agents in the practice of nuclear medicine. *Cancer Biotherapy and Radiopharmaceuticals*.

[35] Grdina DJ, Sigdestad CP (1989). Radiation protectors: the unexpected benefits. *Drug Metabolism Reviews*.

[36] Singh NP, Stephens RE (1997). Microgel electrophoresis: sensitivity, mechanisms, and DNA electrostretching. *Mutation Research*.

[37] Singh NP (2000). Microgels for estimation of DNA strand breaks, DNA protein crosslinks and apoptosis. *Mutation Research*.

[38] Wu LT, Chu CC, Chung JG (2004). Effects of tannic acid and its related compounds on food mutagens or hydrogen peroxide-induced DNA strands breaks in human lymphocytes. *Mutation Research*.

[39] Abdelwahed A, Bouhlel I, Skandrani I (2007). Study of antimutagenic and antioxidant activities of gallic acid and 1,2,3,4,6-pentagalloylglucose from *Pistacia lentiscus*. Confirmation by microarray expression profiling. *Chemico-Biological Interactions*.

[40] Lu Z, Nie G, Belton PS, Tang H, Zhao B (2006). Structure-activity relationship analysis of antioxidant ability and neuroprotective effect of gallic acid derivatives. *Neurochemistry International*.

[41] Yeh C-T, Yen G-C (2006). Induction of hepatic antioxidant enzymes by phenolic acids in rats is accompanied by increased levels of multidrug resistance-associated protein 3 mRNA expression. *Journal of Nutrition*.

[42] Mendiola-Cruz MT, Morales-Ramírez P (1999). Repair kinetics of gamma-ray induced DNA damage determined by the single cell gel electrophoresis assay in murine leukocytes in vivo. *Mutation Research*.

[43] Hofer M, Mazur L, Pospíšil M, Weiterová L, Znojil V (2000). Radioprotective action of extracellular adenosine on bone marrow cells in mice exposed to gamma rays as assayed by the micronucleus test. *Radiation Research*.

[44] Müller W-U, Nüsse M, Miller BM, Slavotinek A, Viaggi S, Streffer C (1996). Micronuclei: a biological indicator of radiation damage. *Mutation Research*.

[45] Henle ES, Linn S (1997). Formation, prevention, and repair of DNA damage by iron/hydrogen peroxide. *Journal of Biological Chemistry*.

[46] Kubo I, Masuoka N, Ha TJ, Shimizu K, Nihei K-I (2010). Multifunctional antioxidant activities of alkyl gallates. *Open Bioactive Compounds Journal*.

[47] Sroka Z, Cisowski W (2003). Hydrogen peroxide scavenging, antioxidant and anti-radical activity of some phenolic acids. *Food and Chemical Toxicology*.

[48] Yen G-C, Duh P-D, Tsai H-L (2002). Antioxidant and pro-oxidant properties of ascorbic acid and gallic acid. *Food Chemistry*.

